# Effect of Average Relative Humidity on Epistaxis

**DOI:** 10.7759/cureus.36063

**Published:** 2023-03-13

**Authors:** Shin Matsumoto, Ryota Ishii, Chihiro Kiuchi, Kotaro Osawa, Masahiro Adachi, Rieko Ii, Masahiro Nakayama, Shuho Tanaka, Keiji Tabuchi

**Affiliations:** 1 Otolaryngology - Head and Neck Surgery, University of Tsukuba, Tsukuba, JPN; 2 Biostatistics, University of Tsukuba, Tsukuba, JPN; 3 Otolaryngology, Tsukuba University Hospital Mito Medical Center, Mito, JPN; 4 Otolaryngology, Tsukuba Gakuen Hospital, Tsukuba, JPN

**Keywords:** nasal mucosa, generalized linear mixed model, climate, humidity, epistaxis

## Abstract

Background

Epistaxis is a very common symptom. The occurrence of epistaxis may be affected by dry environments, but there are some differences among previous reports and this view is controversial.

Objective

We investigated the relationship between the number of epistaxes and daily average relative humidity.

Methods

Data on patients with epistaxis between March 2011 and February 2021 were collected from two hospitals. The daily average relative humidity was examined, and the change in the number of patients with epistaxis due to humidity was investigated using a generalized linear mixed model.

Results

A total of 4184 cases of epistaxis were identified. The number of epistaxis cases per day was significantly associated with the daily average relative humidity (p < 0.001). One percent increment in average relative humidity decreases the number of epistaxis cases per day by 1.1%.

Conclusion

A negative correlation was found to exist between daily average relative humidity and occurrences of epistaxis.

## Introduction

Epistaxis is one of the most common complaints in otolaryngology. Although the detailed pathological causes of epistaxis are unknown, it is commonly believed that epistaxis occurs during the colder, drier months [1]. Several papers reported the relationship between the incidence of epistaxis and a number of environmental factors, including season, temperature, and humidity [1-7]. Although the results varied, most reports commonly supported a negative correlation between the average humidity and the incidence of epistaxis [1,2,4-7]. However, there have been no detailed reports on the extent to which each percentage change in humidity affects epistaxis. The purpose of this study is to investigate the occurrence of epistaxis and the effects of humidity using a large dataset.

## Materials and methods

Patients

The subjects of this study were all patients at Tsukuba University Hospital Mito Medical Center, Mito Kyodo General Hospital, Mito, Japan (hereinafter referred to as Hospital A) and Tsukuba Gakuen Hospital, Tsukuba, Japan (Hospital B), two important acute care hospitals in Ibaraki Prefecture, Japan. The patients were diagnosed with epistaxis from March 2011 to February 2021. The data items collected from each hospital included disease name, age, sex, and date of diagnosis. For statistical analysis, the date of diagnosis was considered as the date of onset of epistaxis.

The study was carried out in accordance with the tenet of the Declaration of Helsinki and the work was compliant with the Strengthening the Reporting of Observational Studies in Epidemiology (STROBE) statement. The institutional review boards of the respective participating institutions approved this study (Tsukuba Clinical Research & Development Organization, approval number: R04-027). By opting out, the institutional review board exempted this retrospective study from the requirement of obtaining informed consent from the individual participants.

Meteorological data

The average relative humidity values analyzed in this study were obtained from the Japan Meteorological Agency website (https://www.jma.go.jp/jma/index.html). The average relative humidity data was adopted for each hospital area. The number of days with the same humidity (%) over a 10-year period was counted. In addition, the number of patients with epistaxis who visited on days with the same humidity (%) was also counted.

Statistical analysis

Data were presented as number (proportion) for categorical variables, and as median (range) for continuous variables. A generalized linear mixed model with Poisson distribution and log link function was used to explore the association between the incidence of epistaxis and average relative humidity, after adjusting for the number of days with the same humidity. In the model, the response variable was the number of patients per day and the fixed effect was the average relative humidity. The hospital was used as a random effect. All statistical analyses were performed with SAS version 9.4 (SAS Institute, Inc, Cary, NC).

## Results

Patient characteristics

Overall, 4184 cases of epistaxis at the participating centers were enrolled in this study. The median age of the patients was 63 years (range: 0 to 97 years). Of the patients, 2520 (60.2%) were male and 1664 (39.8%) were female. The sex ratio and median age were comparable between the two facilities (Table 1).

**Table 1:** Characteristics of patients with epistaxis at participating facilities

		Hospital A		Hospital B		Total	
		(N)	(%)	(N)	(%)	(N)	(%)
Total		2059		2125		4184	
Sex							
	Female	824	(40.0)	840	(39.5)	1664	(39.8)
	Male	1235	(60.0)	1285	(60.5)	2520	(60.2)
Age							
	Median	65		60		63	
	Range	0-97		0-97		0-97	

Effects of humidity on epistaxis

**Figure 1:**
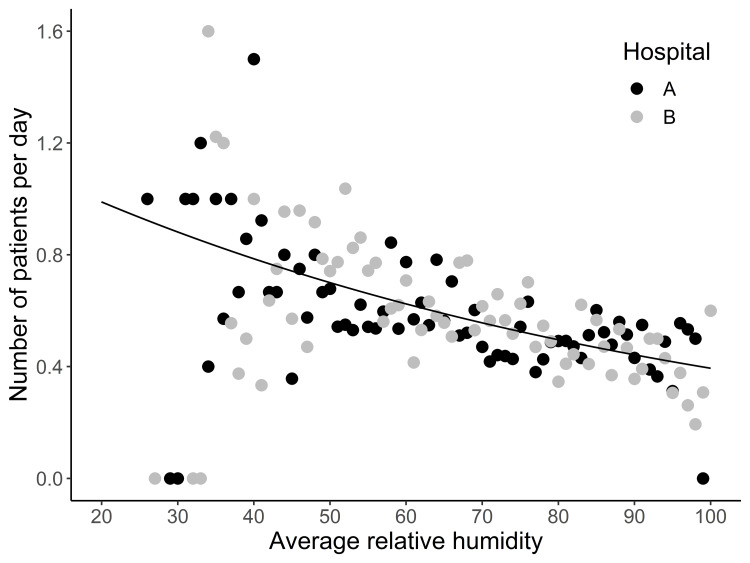
Scatter plot for average relative humidity vs. the number of patients per day: based on models with support using a generalized linear mixed model (GLMM) analysis

The number of epistaxis cases per day was significantly associated with the daily average relative humidity (regression coefficient: -0.0115, p < 0.001). One percent increment in average relative humidity decreased the number of epistaxis cases per day by 1.1% (Figure 1, Table 2).

**Table 2:** Regression coefficients in generalized linear mixed model (GLMM) analysis β, regression coefficient; SE, standard error

	β	SE	p value
intercept	0.2191	0.0816	0.227
average relative humidity	-0.0115	0.0011	<0.0001

## Discussion

In this study, we observed the negative correlation between daily average relative humidity and the number of patients with epistaxis by examining a large amount of data from two hospitals over 10 years. One percent increment in average relative humidity decreased the number of epistaxis cases per day by 1.1%. That is, a 10% increase in average relative humidity resulted in a 10.5% decrease in the number of patients with epistaxis, and a 20% increase resulted in a 19.8% decrease.

Several studies have investigated the relationship between humidity and epistaxis [1-7]. The majority of studies showed a negative correlation between humidity and epistaxis, and our study supported these findings [1,2,4-7]. In addition, this is the first report to investigate the risk of epistaxis for every 1% increment of humidity. Min et al. retrospectively reviewed the medical records of 350 patients with epistaxis at an urban tertiary medical center in Korea for one year and found that a decrease in mean relative humidity was associated with an increase in the number of epistaxis patients [4]. On the other hand, Sowerby’s study, conducted in Alberta, Canada, found no correlation between average humidity and the number of epistaxes [3]. The results may vary depending on the geographic and climatic characteristics of where the study was conducted. McMullin et al. showed a negative correlation between humidity and epistaxis in Saint John, New Brunswick, Canada, a region that is very humid and warm in the summer and very dry and cold in the winter [1]. Regarding the difference from Sowerby’s study, McMullin et al. hypothesize that the variance in epistaxis presentation is more pronounced in areas with distinct seasonal patterns. Regional differences in nasal cavity shape are also known [8] and may contribute to regional differences in results. Our region belongs to the humid subtropical climate of the Köppen climate classification [9]. A humid subtropical climate is characterized by relatively high rainfall throughout the year and large annual variations in temperature. This is the first report from a region with a humid subtropical climate.

Epistaxis has a bimodal age distribution, being more common in children under the age of 10 and adults in their 70s, and occurs more frequently in males [10]. In this study, the male-to-female ratio was three to two. Although this proportion is difficult to generalize, it is common among the reports that it is less common in females [1-7]. The primary female sex hormones, estrogens, are studied as hormonal therapy for epistaxis in hereditary hemorrhagic telangiectasia [11]. One of the effects of estrogens on the coagulation system is the elevation of fibrinogen [12]. Estrogen therapy has been suggested to cause squamous metaplasia of the nasal mucosa [13]. Participation of estrogen is one of the hypotheses for why epistaxis is less common in females, but the detailed relationship is unknown. The population in this study was older, and other factors, such as occupation and smoking, may also have influenced the sex difference.

It is difficult to identify a pathophysiological explanation as to why a decrease in humidity increases the risk of epistaxis. The nasal gland system, stimulated by dry air, protects the lower airways by humidifying the air by producing a variety of secretions [14]. The venous sinuses of the Kieselbach plexus, known to cause many epistaxes, have been observed to vary with the temperature, humidity, and carbon dioxide concentration of the inspired air [14]. Several studies have reported that the administration of dry air into the intranasal cavity reduced intranasal humidity, elevated resistance of the nasal cavity, and decreased mucociliary movements [15,16]. This suggests that the decrease in average relative humidity will reduce intranasal humidity. Additionally, a possible explanation is that the increased frictional resistance of the mucosal surface by the administration of dry air may damage the nasal mucosa and affect epistaxis. However, the underlying mechanism of epistaxis caused by low humidity is unclear, and further research is needed.

In this study, due to the large sample size, we could not review individual charts; therefore, cases of varying severity were included. Information on treatment was not available in the database used for the study. The date of diagnosis of epistaxis was investigated; however, the date of occurrence and the date of diagnosis may not necessarily be the same. Moreover, although our study included the most important acute care hospitals in the region, the extent to which the results of this study can be generalized to other countries with different environments is limited. Despite these limitations, this study provides strong support for the hypothesis that dryness is a risk factor for epistaxis. Our result may be useful for patients who are prone to epistaxis and additional research is needed.

## Conclusions

In this study of a large population in a specific area belonging to the humid subtropical climate, there was a strong negative correlation between daily average relative humidity and the number of patients diagnosed with epistaxis in selected hospitals. This result may be related to increased intranasal resistance and decreased mucociliary movement due to dry air. This result supports the hypothesis that dryness is a risk for epistaxis.
